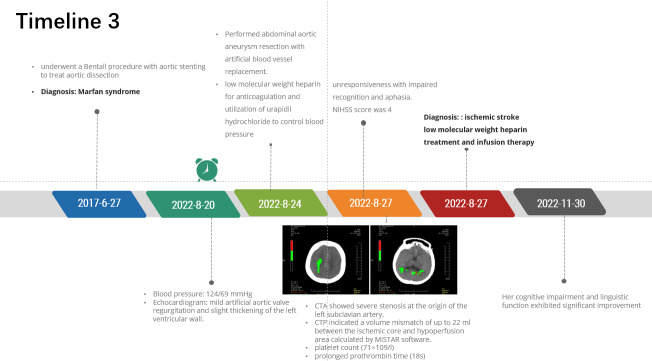# Acute ischemic stroke with a diagnosis of Marfan syndrome: A report of 3 cases in multifaceted settings

**DOI:** 10.1097/MD.0000000000037924

**Published:** 2024-05-10

**Authors:** Licong Chen, Yuan Liu, Jianhua Jiang, Qi Fang, Quanquan Zhang

**Affiliations:** a Department of Neurology, The First Affiliated Hospital of Soochow University, Suzhou, Jiangsu, China; b Department of Neurology, Suzhou Ninth People’s Hospital, Suzhou, Jiangsu, China.

**Keywords:** case report, infective endocarditis, ischemic stroke, Marfan syndrome, thrombolysis

## Abstract

**Rationale:**

Marfan syndrome (MFS), which is a dominantly inherited connective tissue disease resulting from a mutation in the *FBN1* gene, exhibits variable manifestations affecting the cardiovascular, musculoskeletal, ophthalmologic, and pulmonary systems. Notably, neurologic deficiency, which involves ischemic or hemorrhagic stroke, is a rare but severe manifestation. The safety of rt-PA treatment for ischemic stroke caused by MFS is still under discussion.

**Patient concerns:**

In the current report, we discuss 3 atypical MFS cases presented as acute ischemic stroke, compared to those exhibiting cardiovascular and musculoskeletal abnormalities.

**Diagnoses:**

Three patients were diagnosed with acute ischemic stroke accompanied by MFS based on clinical manifestations, imaging examinations, and genetic testings.

**Interventions:**

The first case underwent intravenous thrombolytic therapy with rt-PA, the second case received antiplatelet therapy, and the third case received anticoagulant therapy and perfusion therapy.

**Outcomes:**

The neurologic deficiency of all three patients showed improvement upon discharge, and there were no symptoms of recurrence observed during the follow-up period.

**Lessons subsections:**

MFS is a rare etiology in young people with embolic stroke of undetermined source. Physicians should take MFS into consideration when they observe the characteristic symptoms during a consultation. The potential pathogenesis of ischemic stroke secondary to MFS may include cardio-embolism, arterial dissection, and hypoperfusion. Although intravenous thrombolysis is a promising therapy to treat acute ischemic stroke, further examinations should be conducted to rule out contraindications in patients with a suspicion of MFS.

## 1. Introduction

Marfan syndrome (MFS) is a hereditary connective tissue disorder resulting from a mutation in the fibrillin 1 (*FBN1*) gene located on chromosome 15, characterized by ocular, skeletal, and cardiovascular symptoms as typical manifestations^.[1]^ The morbidity and mortality of MFS are mainly related to complications of the cardiovascular system. MFS patients with severe and chronic valvulopathy resulting in regurgitation may develop left ventricular and atrial dysfunction, aortic dilatation, aortic root aneurysm, and infective endocarditis (i.e.). The diagnosis of MFS is based on the measurement of aortic root standardized by Z-score and genetic testing for *FBN1* mutations.^[2]^

Neurologic symptoms are relatively rare in MFS and generally involve ischemic or hemorrhagic stroke. IS caused by arterial dissection or cardiogenic embolism secondary to i.e. is the most prevalent neurologic complication of MFS.^[3]^

Intravenous recombinant tissue plasminogen activator (rt-PA) is used for the treatment of acute ischemic stroke (AIS) in patients presenting with symptoms within 4.5 hours after stroke onset. However, rt-PA it is not recommended for patients with i.e. due to potential risk of hemorrhagic transformation (class III, level of evidence C).^[4]^ The safety of rt-PA treatment for ischemic cerebral stroke in patients with MFS remains unconfirmed. In this study, we present 3 cases of IS with concomitant MFS in multifaceted settings to investigate the pathogenesis of IS and the etiology of cerebral hemorrhage following intravenous thrombolysis (IVT) in individuals with MFS.

## 2. Case presentation

### 2.1. Case 1

**Figure 1.**
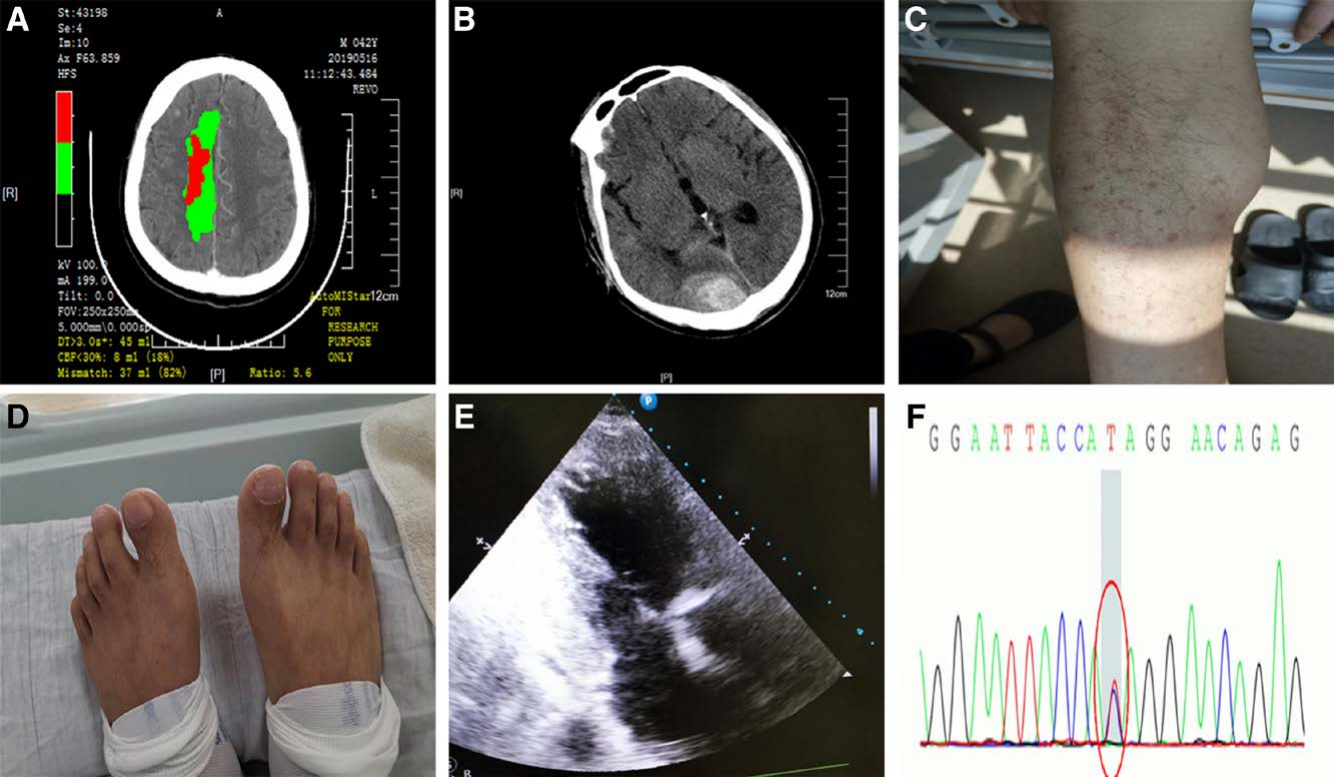
Radiologic findings, physical examination, and gene test results of the patient. (A) CTP showed ischemic perfusion in the blood supply territory of the A4 segment of the right anterior cerebral artery, with a volume mismatch of 82%. (B) Axial cerebral CT scan image reveals hyperdensities in the right occipital lobe along with subarachnoid hemorrhage subsequent to intravenous thrombolysis (IVT). (C) Skin lesions were painless hemorrhagic erythema on both lower limbs. (D) Clubbing of toes. (E) Echocardiogram revealed vegetations in the mitral valve. (F) NGS results for the patient and his mother; both had a heterozygous mutation of c.1177A > G on chr15-48808530. CT = computed tomography, CTP = CT perfusion.

A 42-year-old male patient presented to the emergency department (ED) with a sudden onset of weakness on the left side for 5 hours and 40 minutes. He had a medical history of hypertension. Physical examination revealed a blood pressure reading of 129/71 mm Hg. National Institute of Health Stroke Scale (NIHSS) score was 7 (left facial paralysis = 1, muscle strength of left upper extremity = 4, left lower extremity = 2). The computed tomography (CT) scan of the brain was normal. CT angiography (CTA) showed occlusion in the A4 segment of the right anterior cerebral artery, and CT perfusion (CTP) demonstrated hypoperfusion in the territory supplied by A4, with a volume mismatch of up to 37 mL between the ischemic core and hypoperfusion area calculated by the automatic image analysis function of MISTAR software (Fig. 1A). Considering the diagnosis of AIS and a substantial ischemic penumbra as a tissue window for IVT, intravenous rt-PA was administered 6 hours 20 minutes after symptom onset (see Timeline 1, http://links.lww.com/MD/M270). Two hours after thrombolysis, the patient exhibited an increase in muscle strength of the left extremities to 4/5, accompanied by a reduction in NIHSS score to 1. However, the patient complained of headache in the posterior head and blurred vision. Repeated CT scans revealed multiple hemorrhages in the left cerebellum, left parietal lobe, right occipital and frontal lobes, as well as in the right lateral ventricle and subarachnoid space (Fig. 1B). On the ninth day, the patient presented with fever and complained pain in the interphalangeal joints. The physical examination revealed a peak temperature of 39.2°C as well as erythema and slender limbs with clubbed fingers/toes in the bilateral lower limbs (Fig. 1C and D). A diastolic murmur was detected in the aortic valve auscultation site and a systolic murmur was detected in the mitral site during cardiac auscultation. Viridans streptococci was identified by blood culture. The echocardiogram revealed bilateral aortic valve malformation accompanied by moderate regurgitation, dilatation of the ascending aorta, prolapse of the mitral valve with moderate to severe regurgitation, enlargement of both the left atrium and ventricle, as well as vegetations present in both the mitral and aortic valves (Fig. 1E). Based on these findings, i.e. was strongly suspected. Given the patient maternal history of cerebral hemorrhage, we conducted next-generation sequencing analysis on both the patient and his parent to elucidate potential genetic factors underlying this condition. A heterozygous mutation of c.1177A > G (adenine > guanine) was found in exon 11 of the *FBN1* gene, which was maternally inherited and is related to MFS (Fig. 1F).

Antibacterial teicoplanin was administered for 2 weeks according to drug sensitivity examination. The patient underwent cardiac surgery for vegetation removal and aortic and mitral valve replacement. After discharge from the hospital, the patient was prescribed warfarin (3.125 mg/day peroral). The patient exhibited complete resolution of neurologic deficiency and no recurrence of cerebral infarction when followed up at the outpatient clinic after 5 months. The echocardiogram revealed normal functioning of the artificial valves.

### 2.2. Case 2

**Figure 2.**
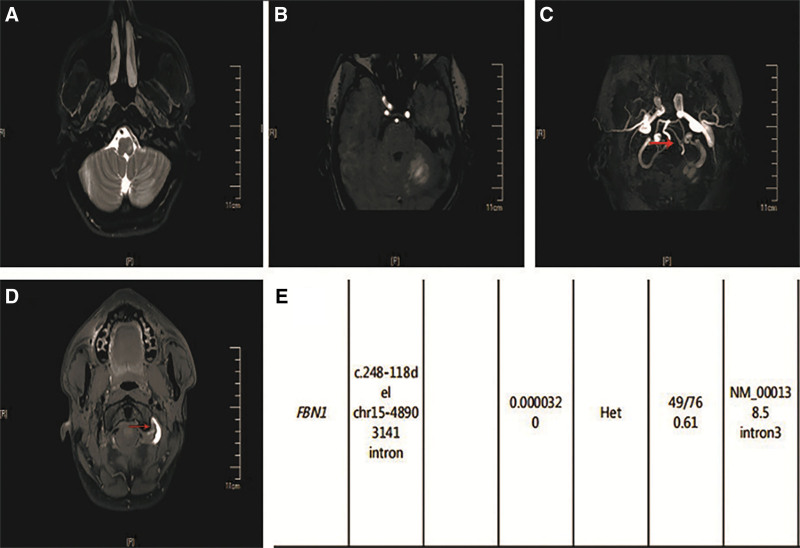
Radiologic findings and gene tests of the patient. (A, B) Hyperintense signals on MRI were detected in the brainstem and left cerebellar hemispheres. (C) High-resolution vessel wall imaging (HRVW) MRI showed fenestration of the V4 segment of the left vertebral artery. (D) HRVW MRI showed vertebral artery dissection of the V3 segment with possible intramural hematoma. (E) Results of NGS for the patient. The patient had a heterozygous mutation on chr15-48903141. MRI = magnetic resonance imaging, NGS = next-generation sequencing.

A 29-year-old male with a tall and slender physique presented to the ED due to a 4-day history of dizziness accompanied by an unsteady gait. The patient had a medical history of compulsory spondylitis and was receiving routine etanercept (50 mg by subcutaneous injection). The physical examination revealed a blood pressure reading of 134/86 mm Hg in the right arm while the patient was in a supine position. Examination of the musculoskeletal system revealed a slender individual measuring 189 cm in height and weighing 63 kg. The NIHSS score of the patient was 1 (ataxia of the left extremity = 1) and he was diagnosed with IS. However, considering the thrombolytic time window, antiplatelet therapy was ultimately administered (see Timeline 2, http://links.lww.com/MD/M271). Magnetic resonance imaging revealed multiple acute cerebral infarcts in the brainstem and bilateral cerebellar hemispheres (Fig. 2A and B). Laboratory tests showed human leukocyte antigen class I molecule B27(+), supporting the diagnosis of ankylosing spondylitis. The routine blood tests, blood coagulation function, serum lipid levels, as well as laboratory and immunologic tests in rheumatology were all normal. Transesophageal echocardiogram showed aortic root enlargement. CTA revealed fenestration of the V4 segment and moderate stenosis in the V3 segment of the left vertebral artery. CTP demonstrated hypoperfusion in the left cerebellar hemisphere. High-resolution vessel wall imaging was conducted, indicating vertebral artery dissection of the V3 segment with possible intramural hematoma and fenestration of the V4 segment (Fig. 2C and D). The neurologic symptoms were considered to be associated with the left vertebral artery dissection. However, the patient had no prior history of stretch exercise, cupping therapy, or other exercises that could induce arterial dissection. Considering the presence of long bone overgrowth, aortic root dilatation, and a history of ankylosing spondylitis, MFS was suspected despite the absence of any family medical history. The patient underwent genetic testing, revealing the presence of a heterozygous mutation in the *FBN1* gene (Fig. 2E). The patient was definitively diagnosed with MFS. He exhibited a remarkable alleviation in clinical symptoms and was discharged with prescriptions for aspirin, clopidogrel and rosuvastatin. The patient exhibited no neurological deficits during the 6-month follow-up conducted at the outpatient clinic.

### 2.3. Case 3

**Figure 3.**
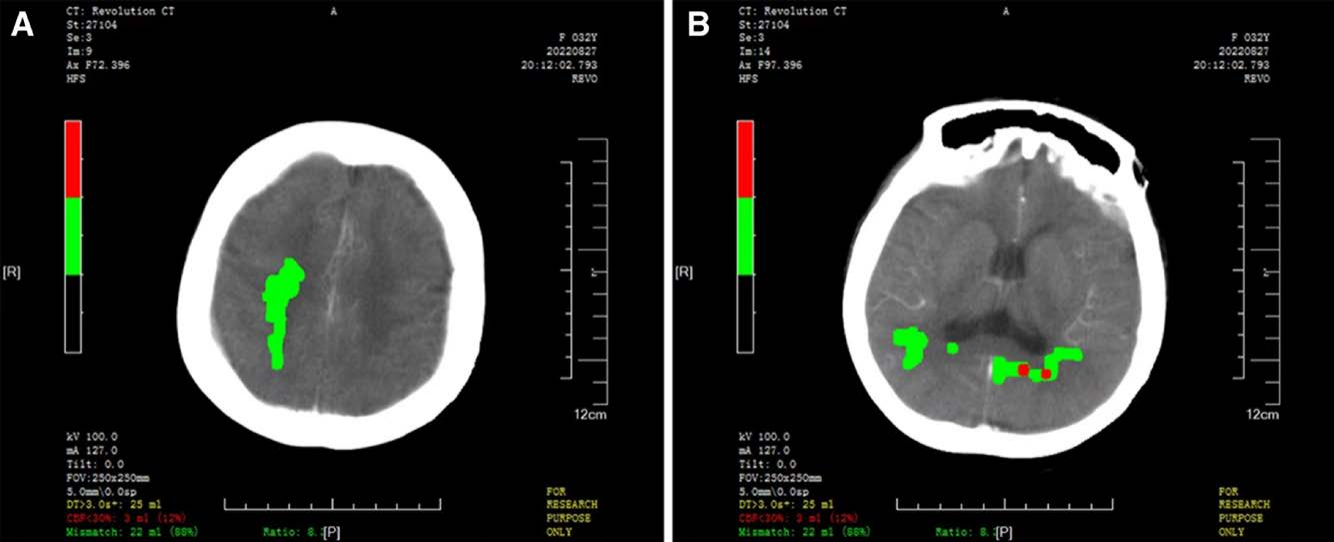
CT perfusion in the acute phase. (A, B) CTP showed a volume mismatch of up to 22 mL between the ischemic core and hypoperfusion area. CT = computed tomography, CTP = CT perfusion.

A 32-year-old female patient was found unresponsive accompanied by impaired recognition and aphasia persisting for 8 hours. She had been admitted to the Department of Cardiac Surgery with abdominal aortic aneurysm (AAA) 7 days prior. She was diagnosed as MFS and underwent a Bentall procedure with aortic stenting to treat aortic dissection 5 years prior. On admission, the patient blood pressure was 124/69 mm Hg. Echocardiogram showed mild artificial aortic valve regurgitation and slight thickening of the left ventricular wall. AAA resection with artificial blood vessel replacement was performed 4 days after admission. Postoperative treatments encompassed administration of low molecular weight heparin for anticoagulation and utilization of urapidil hydrochloride to control blood pressure. Three days after surgery, the patient presented with neurologic symptoms including unresponsiveness with impaired recognition and aphasia. The NIHSS score was 4 (level of consciousness questions = 1, level of consciousness commands = 1, severe aphasia = 2). The patient was unable to receive magnetic resonance imaging examination. CTA showed severe stenosis at the origin of the left subclavian artery. CTP indicated a volume mismatch of up to 22 ml between the ischemic core and hypoperfusion area calculated by MISTAR software (Fig. 3A and B). The examinations mentioned above indicated the hypoperfusion characteristic of the stroke, which was attributed to postoperative treatment for blood pressure control and hemodynamic complications resulting from unclamping-induced hypotension. The patient did not receive IVT treatment due to the lower platelet count (71 × 10^9^/l) and the prolonged prothrombin time (18s). The patient continued to receive low molecular weight heparin treatment, and infusion therapy was also administered in order to enhance cerebral perfusion (see Timeline 3, http://links.lww.com/MD/M272). The patient neurologic status improved, with a decrease in NIHSS score to 0 at discharge. Her cognitive impairment and linguistic function exhibited significant improvement during the 3-month follow-up at the outpatient clinic.

## 3. Discussion

MFS is a systemic connective tissue disorder with a prevalence of 2 to 3/100,000 individuals.^[5]^ Approximately 75% of individuals with MFS manifest an autosomal dominant inherited disorder resulting from a mutation in the *FBN1* gene, while approximately 25% exhibit a de novo pathogenic variant or germline mosaicism of *FBN1*.^[3,5]^ MFS exhibits variable manifestations affecting the cardiovascular, musculoskeletal, ophthalmologic, and pulmonary systems. Notably, cardiovascular symptoms such as aortic root aneurysm and mitral valve prolapse/regurgitation are considered as primary causes of mortality. The diagnosis of MFS in individuals with these symptoms is based on the identification of aortic root enlargement (Z-score ≥ 2.0) by echocardiogram and the detection of *FBN1* gene mutation by next-generation sequencing.

Neurovascular complications of MFS are rare and remain poorly understood. A case-control study involving 13,000 individuals diagnosed with MFS revealed an upward trend in the prevalence of comorbidities such as ischemic stroke, hemorrhagic stroke, and cerebral aneurysms within this population. The estimated morbidity rate of AIS exceeds 2%, while the incidence of cervical carotid dissection is 11.7 times higher.^[6]^ In a retrospective study involving 513 individuals with MFS revealed that the incidence of IS was 3% and that of hemorrhagic stroke was 0.5%.^[7]^ IS may arise from various arterial pathologies including aortic aneurysm, both extra- and intracranial carotid artery dissection, postoperative abnormalities in the aortic root or valves and cardiac valve disease.^[3,6]^ Arterial dissection is the most prevalent etiology of AIS in individuals with MFS.^[8]^ The second case suffered from cerebral artery dissection. Carotid or vertebral artery dissection causes acute cerebral infarction in 2% of cases while accounts for 10% to 25% of IS among young and middle-aged individuals.^[9]^ Intimal tear is a common cause of carotid and vertebral artery dissections. Connective tissue disease, fibromuscular dysplasia and infection are related to an increased risk of spontaneous dissection. About 15% of the individuals with spontaneous dissection of the carotid or vertebral artery have angiographic signs of fibromuscular dysplasia.^[10–12]^ The coexistence of fibromuscular dysplasia and cystic medial necrosis was observed in a reported case with MFS and bilateral cervical artery dissection.^[13]^ Pathological manifestations of MFS usually includes cystic medial necrosis and disruption of elastic fibers, potentially contributing to spontaneous dissection of cerebral arteries independent of aortic dissection.^[7]^ Previously reported cases indicate that patients with MFS are susceptible to ischemic stroke with hemorrhagic transformation caused by cerebral artery dissection.^[14,15]^

IS in the third presented case appeared to be caused by cerebral hypoperfusion associated with blood pressure control and hemodynamic complications due to unclamping hypotension during AAA surgery. Thoracoabdominal aortic aneurysm is largely a degenerative disease (80%) with approximately 15% to 20% secondary to chronic dissection and a minority because of MFS (5%).^[16]^ Reconstructive surgery involving graft placement has been employed for the treatment of AAA. The major risks in surgical AAA repair are massive blood loss, persistent unclamping hypotension, sigmoid colon ischemia and distal artery embolization.^[17]^ The situation can be presented as our third case.

Robert et al reported that cardioembolism plays a secondary role in AIS in MFS patients, just next to arterial dissection.^[8]^ The first case exhibited a cardioembolic etiology since we observed the detachment of bacterial emboli from cardiac valves ex post. However, the patient was treated with intravenous thrombolytic therapy according to clinical presentations, laboratory and radiological findings. Although the patient clinical symptoms were improved, he suffered from intracerebral hemorrhage after thrombolytic treatment. MFS is often complicated by valvular heart disease, which could lead to i.e.. A major complication of i.e. is embolic stroke, resulting from the detachment and migration of embolus originating from a heart valve vegetation to the cerebral vessels. Since the risk of hemorrhage after IVT is about 20%, AIS caused by i.e. is not recommended to receive thrombolytic therapy.^[18]^ The first presented case exhibited hemorrhagic transformation after IVT and was subsequently diagnosed as i.e. Echocardiography was not performed on the patient as we opted for oral consultation to exclude i.e., aiming to minimize the door-to-needle time. It indicates us that examinations such as echocardiography and CTA should be performed in individuals with a medical history of MFS to exclude the presence of infectious endocarditis, aortic coarctation and other complications associated with MFS.

The main clinical features of IS accompanied by MFS reported in recent years are presented in Table 1.^[3,5,14,15,19–24]^ Among the 10 recently reported cases, 5 of them are considered to be cardioembolic while 3 exhibit arterial dissection. Regardless of IVT treatment, 3 of the recently reported cases suffered hemorrhagic transformation. The outcomes of all the reported cases were favorable, as were those of our patients.

**Table 1:** Baseline characteristics of cases of ischemic stroke caused by MFS reported in the literature.

Patient (reference)	Yr	Age/sex	Known MFS	Presentation	Intravenous thrombolysis	Mechanical thrombectomy	Hemorrhagic complications	Possible mechanism of IS	Outcome
1 (Souirti 2011)^[3]^	2011	17/M	No	Right hemiparesis, brachiofacial dominance, hemihypoesthesia, and Broca aphasia	No	No	No	Cardioembolic origin (infectious endocarditis)	Complete improvement
2 (Malek 2019)^[5]^	2019	44/M	Yes	Sudden onset of left-sided weakness and slurred speech	Yes	No	No	Chronic aortic dissection	Near-complete recovery
3 (Maski 2011)^[14]^	2011	19/M	Yes	Severe, throbbing frontal headache, then ataxia and an audible bruit at the base of his skull	No	No	Yes	Dissection within the V4 segment of the right vertebral artery	Complete improvement
4 (Ozawa 2018)^[15]^	2018	32/M	Yes	Right hemiparesis and slurred speech	No	No	No	Dissection of the right middle cerebral artery	Complete improvement
5 (Anton 2006)^[19]^	2006	27/M	Yes	Left-sided mild weakness, dysphasia, right frontal headache	No	No	No	Cardioembolic mechanism (2 bioprosthetic heart valve surgeries and no evidence of aortic or carotid dissection)	Complete improvement
6 (Micelli 2018)^[20]^	2018	16/M	No	Sudden weakness of right arm and leg, dysarthria	No	No	Yes	Embolic stroke (left atrial myxoma)	Improvement
7 (Gójska-Grymajło 2014)^[21]^	2014	34/M	Yes	Sudden onset of double vision and right face and tongue paresthesia	Yes	No	No	Probable cardio-embolic etiology	Complete improvement
8 (Chembala 2012)^[22]^	2012	54/M	Yes	Sudden onset of right arm and leg weakness and slurred speech	Yes	No	Yes	Cardioembolic (poor left ventricular function and prosthetic valves)	Improvement
9 (Zhu 2022)^[23]^	2022	18/M	No	Right hemiparesis, aphasia, and impaired consciousness	Yes	Yes	No	Embolism of the basilar artery that may originate from aortic root aneurysm	Near-complete recovery
10 (Wang 2019)^[24]^	2019	32/M	No	Sudden onset of right arm and leg weakness and slurred speech	Yes	Yes	No	Probable cardioembolic etiology (prior history of definite heart valve replacement)	Improvement

IS = ischemic stroke, M = male, MFS = Marfan syndrome.

We reported 3 cases of MFS complicated with IS, each with a distinct mechanism and requiring different treatment approaches. The limitation of our report is that only 1 patient received thrombolytic therapy, resulting in hemorrhagic transformation; however, the prognosis for this patient remained relatively favorable. The safety of thrombolytic therapy for AIS in patients with MFS remains uncertain, necessitating further research.

## 4. Conclusions

We present 3 cases with atypical MFS diagnosis accompanied with IS compared to those manifest as cardiovascular and musculoskeletal abnormalities. MFS is a rare diagnosis in young people with unexplainable stroke. Physicians should take MFS into consideration when they observe the characteristic symptoms during a consultation. The potential etiologies of stroke in patients with MFS may encompass cardioembolism, arterial dissection and hypoperfusion. Although IVT is a promising therapy to treat IS, further examinations should be conducted to rule out contraindications in patients with a suspected of MFS.

## Acknowledgments

We appreciate the efforts of my colleagues and the support of the patients.

## Author contributions

**Conceptualization:** Qi Fang, Quanquan Zhang.

**Funding acquisition:** Quanquan Zhang.

**Investigation:** Licong Chen, Yuan Liu, Jianhua Jiang.

**Methodology:** Yuan Liu, Jianhua Jiang.

**Resources:** Quanquan Zhang.

**Validation:** Qi Fang.

**Visualization:** Qi Fang.

**Writing – original draft:** Licong Chen.

## Supplementary Material

**Figure:**
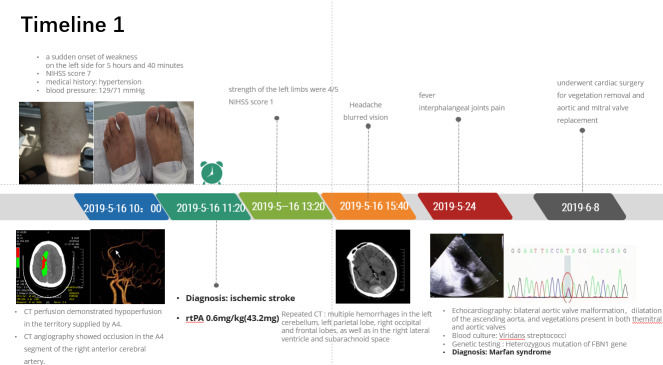


**Figure:**
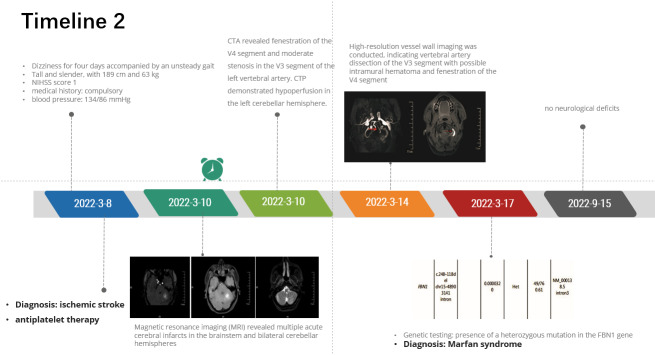


**Figure:**